# Matching depression management to severity prognosis in primary care: results of the Target-D randomised controlled trial

**DOI:** 10.3399/BJGP.2020.0783

**Published:** 2021-01-12

**Authors:** Susan Fletcher, Patty Chondros, Konstancja Densley, Elizabeth Murray, Christopher Dowrick, Amy Coe, Kelsey Hegarty, Sandra Davidson, Caroline Wachtler, Cathrine Mihalopoulos, Yong Yi Lee, Mary Lou Chatterton, Victoria J Palmer, Jane Gunn

**Affiliations:** Department of General Practice, Melbourne Medical School, University of Melbourne, Melbourne, Australia.; Department of General Practice, Melbourne Medical School, University of Melbourne, Melbourne, Australia.; Department of General Practice, Melbourne Medical School, University of Melbourne, Melbourne, Australia.; Department of General Practice, Melbourne Medical School, University of Melbourne, Melbourne, Australia; professor of eHealth and primary care, Research Department of Primary Care and Population Health, University College London, London, UK.; Department of General Practice, Melbourne Medical School, University of Melbourne, Melbourne, Australia; professor of primary medical care, Department of Health Services Research, University of Liverpool, Liverpool, UK.; Department of General Practice, Melbourne Medical School, University of Melbourne, Melbourne, Australia.; Department of General Practice, Melbourne Medical School, University of Melbourne; director, Centre for Family Violence Prevention, The Royal Women’s Hospital, Melbourne, Australia.; Department of General Practice, Melbourne Medical School, University of Melbourne, Melbourne, Australia.; Department of General Practice, Melbourne Medical School, University of Melbourne, Melbourne, Australia; family medicine resident, Department of General Practice and Primary Care, Karolinska Institutet, Solna, Sweden.; Deakin Health Economics, Institute for Health Transformation, Deakin University, Geelong, Australia.; Deakin Health Economics, Institute for Health Transformation, Deakin University, Geelong; honorary fellow, School of Public Health, University of Queensland, Brisbane; health economist, Policy and Epidemiology Group, Queensland Centre for Mental Health Research, Brisbane, Australia.; Deakin Health Economics, Institute for Health Transformation, Deakin University, Geelong, Australia.; Department of General Practice, Melbourne Medical School, University of Melbourne, Melbourne, Australia.; Faculty of Medicine, Dentistry and Health Sciences, University of Melbourne; chair of primary care research, Department of General Practice, Melbourne Medical School, University of Melbourne, Melbourne, Australia.

**Keywords:** mental health, primary health care, general practice, randomised controlled trial, clinical prediction rule

## Abstract

**Background:**

Mental health treatment rates are increasing, but the burden of disease has not reduced. Tools to support efficient resource distribution are required.

**Aim:**

To investigate whether a person-centred e-health (Target-D) platform matching depression care to symptom severity prognosis can improve depressive symptoms relative to usual care.

**Design and setting:**

Stratified individually randomised controlled trial in 14 general practices in Melbourne, Australia, from April 2016 to February 2019. In total, 1868 participants aged 18–65 years who had current depressive symptoms; internet access; no recent change to antidepressant; no current antipsychotic medication; and no current psychological therapy were randomised (1:1) via computer-generated allocation to intervention or usual care.

**Method:**

The intervention was an e-health platform accessed in the GP waiting room, comprising symptom feedback, priority-setting, and prognosis-matched management options (online self-help, online guided psychological therapy, or nurse-led collaborative care). Management options were flexible, neither participants nor staff were blinded, and there were no substantive protocol deviations. The primary outcome was depressive symptom severity (9-item Patient Health Questionnaire [PHQ-9]) at 3 months.

**Results:**

In intention to treat analysis, estimated between- arm difference in mean PHQ-9 scores at 3 months was −0.88 (95% confidence interval [CI] = −1.45 to −0.31) favouring the intervention, and −0.59 at 12 months (95% CI = −1.18 to 0.01); standardised effect sizes of −0.16 (95% CI = −0.26 to −0.05) and −0.10 (95% CI = −0.21 to 0.002), respectively. No serious adverse events were reported.

**Conclusion:**

Matching management to prognosis using a person-centred e-health platform improves depressive symptoms at 3 months compared to usual care and could feasibly be implemented at scale. Scope exists to enhance the uptake of management options.

## INTRODUCTION

Despite significant investment in improving access to care,^1^ depression remains a leading contributor to the burden of disease and constitutes a significant public health issue.^2^ This in part reflects suboptimal targeting of care, with both over- and under-treatment occurring.^1^^–^^3^ Treatment guidelines and policy initiatives have aimed to address this mismatch by encouraging provision of the least intensive intervention that is likely to be effective for an individual (an approach known as stepped care).^4^^,^^5^ However, there is currently no consensus as to how the appropriate level of intervention intensity is identified. Therefore, building the evidence base to support the implementation of stepped care is key to reducing the time and resources currently required to identify an individual’s mental needs and match them to care accordingly.^6^^–^^8^

Such evidence is particularly important for primary care, where the majority of depression care is delivered.^9^^,^^10^ Currently, GPs rely mostly on clinical judgement when allocating depression care, which can be a time-consuming and inconsistent process.^8^ This is in contrast to other areas of medicine where a range of clinical prediction tools (CPTs) are available to streamline systematic decision making,^11^^–^^15^ although there is increasing recognition that such tools must be user-friendly and action-oriented in order to be successfully translated in routine practice.^16^ To address this gap the authors developed a CPT that uses self-reported biopsychosocial data to classify individuals into one of three prognostic groups based on the predicted severity of their depressive symptoms in 3 months’ time (minimal/mild, moderate, or severe).^17^ The CPT was then embedded into an e-health platform^18^ (henceforth referred to as the Target-D platform), which was designed using the principles of motivational interviewing^19^ and psychologically-driven goal modelling^20^ to deliver a person-centred approach to depression care.^18^ The Target-D platform provides patients with feedback on their responses, an opportunity to reflect on their mental health priorities and motivation to change, and a management option matched to their severity prognosis.

**Table:** How this fits in

Depression is a leading contributor to the global burden of disease and a significant problem in primary care, where it is typically identified and managed. Stepped care approaches are recommended but difficult to implement in routine care, due in part to a lack of effective tools to guide GPs in matching intervention intensity to patient need. Therefore, a clinical prediction tool was developed, which was embedded into a person-centred e-health platform, that matches depression management options to symptom severity prognosis. This randomised controlled trial showed using this platform results in greater improvement in depressive symptoms at 3 months compared to usual care. This approach could be implemented in routine care to support more efficient and effective depression care without adding to GPs’ workload.

In the Target-D randomised controlled trial (RCT), the primary aim was to investigate whether a complex intervention comprising the Target-D platform and matched management options for primary care patients with depressive symptoms improved depressive symptoms at 3-month follow-up, relative to usual care plus attention control (UC+). Secondary aims were to test for an intervention effect at 12 months overall, and within prognostic groups at both 3 and 12 months.

## METHOD

### Study design

This is a stratified individual RCT, enrolling primary care patients who screened positive for depressive symptoms (see protocol for details^21^). The intervention period lasted 3 months. No substantive changes to the published protocol were made.

### Participants

Research assistants (RAs) recruited participants from the waiting rooms of 14 general practices in metropolitan Melbourne, Australia (Supplementary Appendix S1 describes practice characteristics). Adults aged 18–65 years were invited to complete an eligibility survey on an iPad, and were eligible if they reported: current depressive symptoms (≥2 on the 2-item version of the Patient Health Questionnaire [PHQ-2]^22^); no self-reported change to antidepressant medication in the past month; had access to the internet; and sufficient written English to follow an internet-based cognitive behavioural therapy (iCBT) programme. Patients reporting current use of antipsychotic medication or receipt of psychological therapy (online or face-to-face) were ineligible.

Randomisation occurred after participants provided informed consent and completed baseline measures (including items required for the CPT), all integrated with the Target-D platform on a purpose-built website accessible on any internet-enabled device.

### Interventions

All participants received an automated email encouraging them to speak with their GP if they had concerns about their mental health and providing contact details for community-based services (for example, crisis support lines).

#### Intervention arm

Individuals received CPT feedback, set priorities, and received a management option matched to the prognostic group (see Supplementary Appendix S2). Briefly:

- Minimal/mild: myCompass online programme,^23^ a CBT-based self-help resource where participants could choose from 15 modules (such as, ‘Tackling Unhelpful Thinking’ and ‘Communicating Clearly’).
- Moderate: Worry and Sadness course of the This Way Up iCBT programme,^24^ which required participants to work through six lessons in sequence.
- Severe: nurse-led collaborative care including up to eight contacts (over telephone or in person) with a trained research nurse to develop and implement a tailored depression management plan in conjunction with their GP.^25^^–^^29^

#### Control arm

Individuals received UC+ telephone call from an RA about trial involvement and to seek views about research participation.

### Randomisation and blinding

Participants were randomly assigned to a trial arm (1:1, stratified by practice and prognostic group) using a computer- generated biased-coin algorithm with an imbalance intolerance of three embedded in the purpose-built website (see Supplementary Appendix S3).

Due to the nature of the intervention, participants could not be blinded to their allocated management option. Staff involved in intervention delivery (for example, RAs discussing management options or nurses delivering collaborative care) were also unblinded. GPs were notified only of participants allocated to collaborative care, with no emergency unblinding required. RAs responsible for contacting participants at follow-up were blinded to trial arm and prognostic group. All analyses were conducted and discussed while statisticians and study investigators remained blind to trial arm allocation.

### Outcomes

Data were collected primarily via online survey at baseline and at 3- and 12-months post-randomisation. At each time-point, non-responders received phone, text, and/or email reminders and were offered alternative options for completion (such as via hard copy or phone). At trial enrolment, participants provided information on demographic (age, sex, education, and employment) and clinical characteristics relevant to trial exclusion criteria.

The primary outcome was depressive symptom scores at 3 months, assessed using the 9-item Patient Health Questionnaire (PHQ–9).^30^

Secondary outcomes included: depressive symptom severity at 12 months; anxiety symptom severity assessed using the 7-item Generalised Anxiety Disorder scale (GAD-7);^31^ mental health self-efficacy measured using the Mental Health Self- Efficacy Scale (MHSES);^32^ and quality of life using the Assessment of Quality of Life Instrument (AQoL-8D)^33^ at 3 and 12 months.

### Sample size

For the primary hypothesis, 1320 participants (*n* = 660 per arm) provided 90% power at 5% significance two-tailed alpha to detect a standardised mean difference (SMD) of 0.2 in depressive symptoms at 3 months, assuming 20% attrition over 12 months of the follow-up period. For secondary hypotheses, the authors had 80% power to detect a between-arm SMD of 0.2 in depressive symptoms in the minimal/mild group and 0.5 within the moderate and severe groups, respectively. Calculations assumed 70% (*n* = 924) of participants would be in the minimal\mild group and 15% (*n* = 198) in each of the moderate and severe groups.

### Statistical methods

All analyses were pre-defined in the statistical analysis plan^34^ and conducted using Stata (version 15). Main analyses employed an intention to treat (ITT) approach, where all participants were analysed in the trial arm to which they were allocated. Differences in mean outcomes between trial arms (intervention effect) were estimated with linear mixed- effects models, using restricted maximum likelihood with random intercepts for individuals. Stratification factors (practice or prognostic group) and time (baseline, 3, and 12 months) were included as fixed effects, with a two-way interaction between arm and time, except at baseline where trial-arm means were constrained to be equal. Similar mixed effects analysis was conducted for each prognostic group. Sensitivity analyses included random-effects for nurse in the severe group and assessed the robustness of the missing data assumption (see Supplementary Appendix S4). Complier average causal effect (CACE) analysis^35^ used a two stage-least squares instrumental regression with trial arm used as the instrumental variable for adherence to treatment.^34^

## RESULTS

**Figure 1.**
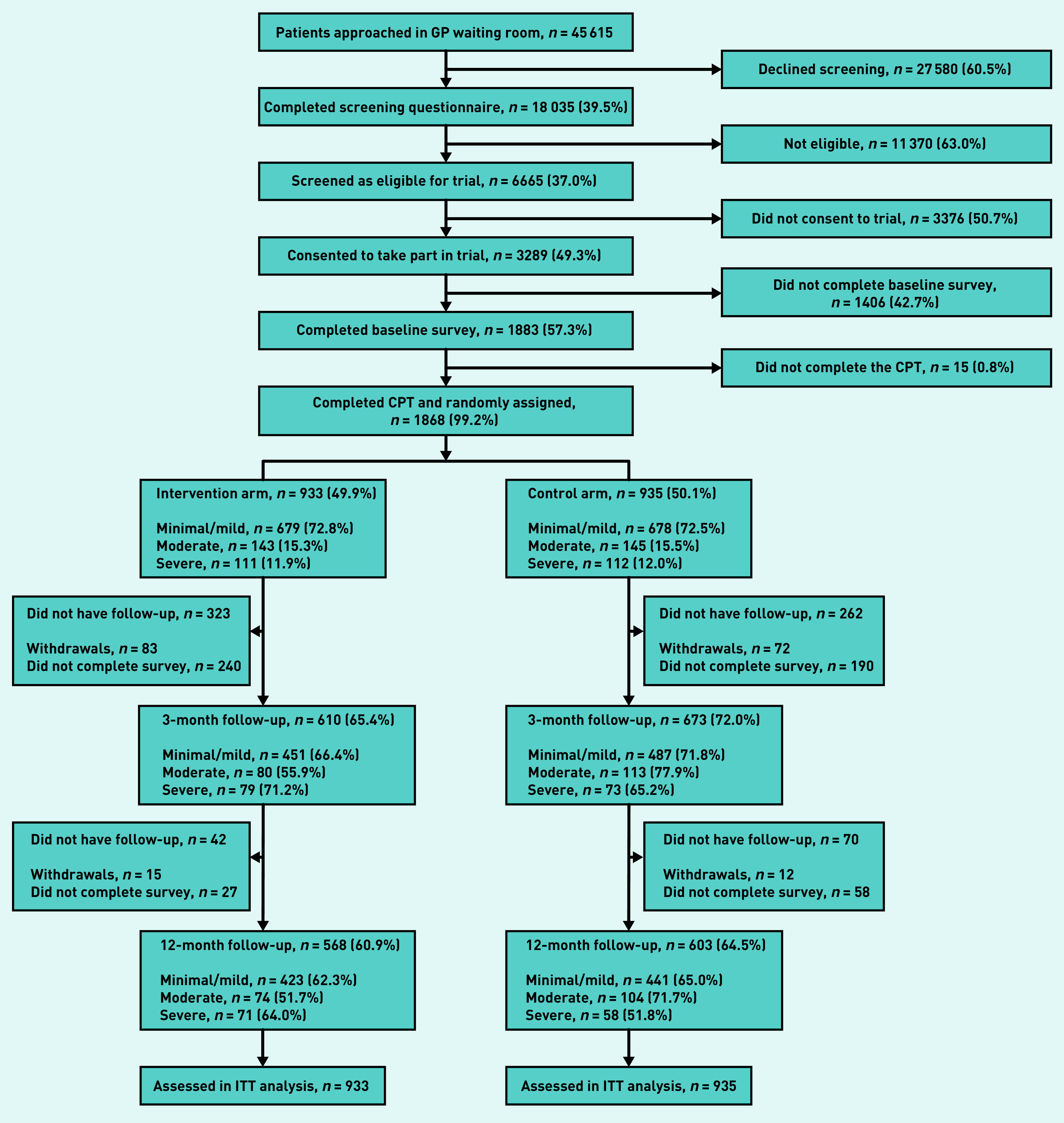
***Participant flow through the trial. Denominators used to calculate the percentage with follow-up at 3- and 12-months are the total number of participants randomised overall and within each prognostic group. None of the withdrawals requested that their data be withdrawn prior to the statistical analysis. CPT = clinical prediction tool. ITT = intention to treat.***

Figure 1 shows the trial profile. There were 1868 participants in total (*n* = 1270 female; *n* = 590 male; *n* = 8 other; mean age 35.5 [standard deviation = 12.1] years). The CPT classified 1357 (72.6%) participants to the minimal/mild group, 288 (15.4%) to the moderate group and 223 (11.9%) to the severe group. Some differential attrition was evident, with retention higher in the control arm overall (and within the minimal/mild and moderate prognostic groups). In the severe group, retention was higher in the intervention arm. Participants in the two trial arms were similar, overall and within prognostic groups (see Table 1 and Supplementary Appendix S1).

**Table 1.** Baseline characteristics of participant according to trial arm, in total and stratified by prognostic group, *N* = 1868

	**All participants, *N* = 1868**		**Prognostic group**					

			**Minimal/mild, *n* = 1357**		**Moderate,**	***n* = 288**	**Severe, *n* = 223**	
	
	**Intervention, *n* = 933, mean (SD)**	**Control, *n* = 935, mean (SD)**	**Intervention, *n* = 679, mean (SD)**	**Control, *n* = 678, mean (SD)**	**Intervention, *n* = 143, mean (SD)**	**Control, *n* = 145, mean (SD)**	**Intervention, *n* = 111, mean (SD)**	**Control, *n* = 112, mean (SD)**
**Age, years**	35.5 (12.1)	35.6 (12.1)	35.2 (11.7)	35.5 (11.8)	36.0 (13.1)	35.5 (12.5)	36.3 (13.4)	36.5 (13.1)

**Depressive symptom severity (PHQ-9)**	9.2 (5.8)	9.3 (5.7)	6.4 (3.4)	6.6 (3.4)	14.2 (2.3)	13.9 (2.4)	19.7 (3.5)	19.6 (3.6)

**Anxiety symptom severity (GAD-7)^a^**	8.6 (5.3)	8.7 (5.1)	6.7 (4.2)	7.0 (4.2)	11.6 (4.1)	11.4 (4.5)	15.9 (3.7)	14.8 (4.7)

**Mental health self-efficacy (MHSES)^a^**	38.1 (12.2)	37.4 (12.1)	42.2 (10.5)	41.4 (10.5)	30.2 (9.3)	30.5 (9.0)	24.4 (9.8)	23.1 (8.8)

**Quality of life (AQoL-8D)^a^**	0.6 (0.2)	0.6 (0.2)	0.6 (0.2)	0.6 (0.2)	0.4 (0.1)	0.4 (0.1)	0.3 (0.1)	0.3 (0.1)

	***n* (%)**	***n* (%)**	***n* (%)**	***n* (%)**	***n* (%)**	***n* (%)**	***n* (%)**	***n* (%)**

**Sex**								
Male	313 (33.5)	277 (29.6)	225 (33.1)	191 (28.2)	52 (36.4)	45 (31.0)	36 (32.4)	41 (36.6)
Female	617 (66.1)	653 (69.8)	453 (66.7)	485 (71.5)	90 (62.9)	98 (67.6)	74 (66.7)	70 (62.5)
Other	3 (0.3)	5 (0.5)	1 (0.1)	2 (0.3)	1 (0.7)	2 (1.4)	1 (0.9)	1 (0.9)

**Highest level of education completed**								
Year 11 or less	109 (11.7)	112 (12.0)	65 (9.6)	68 (10.0)	21 (14.7)	20 (13.8)	23 (20.7)	24 (21.4)
Year 12 or equivalent	136 (14.6)	146 (15.6)	93 (13.7)	94 (13.9)	22 (15.4)	31 (21.4)	21 (18.9)	21 (18.8)
Certificate/diploma	211 (22.6)	230 (24.6)	140 (20.6)	169 (24.9)	35 (24.5)	30 (20.7)	36 (32.4)	31 (27.7)
Bachelor’s degree or higher	477 (51.1)	447 (47.8)	381 (56.1)	347 (51.2)	65 (45.5)	64 (44.1)	31 (27.9)	36 (32.1)

**Employment status**								
Employed/working for profit or pay	686 (73.5)	667 (71.3)	513 (75.6)	509 (75.1)	99 (69.2)	89 (61.4)	74 (66.7)	69 (61.6)
Unemployed	92 (9.9)	119 (12.7)	64 (9.4)	66 (9.7)	17 (11.9)	30 (20.7)	11 (9.9)	23 (20.5)
Neither working nor looking for work	155 (16.6)	149 (15.9)	102 (15.0)	103 (15.2)	27 (18.9)	26 (17.9)	26 (23.4)	20 (17.9)

**Receiving benefit or disability support^a^**	100 (11.6)	133 (15.4)	49 (7.9)	79 (12.7)	24 (17.1)	30 (21.9)	27 (26.2)	24 (22.6)

**History of depression**	582 (62.4)	593 (63.4)	341 (50.2)	348 (51.3)	130 (90.9)	137 (94.5)	111 (100.0)	108 (96.4)

**Long term illness**	245 (26.3)	270 (28.9)	124 (18.3)	129 (19.0)	56 (39.2)	70 (48.3)	65 (58.6)	71 (63.4)

**Self-rated health**								
Excellent/very good/good	732 (78.5)	729 (78.0)	596 (87.8)	589 (86.9)	94 (65.7)	94 (64.8)	42 (37.8)	46 (41.1)
Fair/poor	201 (21.5)	206 (22.0)	83 (12.2)	89 (13.1)	49 (34.3)	51 (35.2)	69 (62.2)	66 (58.9)

**Live alone**	130 (13.9)	109 (11.7)	80 (11.8)	62 (9.1)	30 (21.0)	26 (17.9)	20 (18.0)	21 (18.8)

**Manage on available income**								
Easily/not too bad/difficult some of the time	832 (89.2)	817 (87.4)	643 (94.7)	640 (94.4)	121 (84.6)	111 (76.6)	68 (61.3)	66 (58.9)
Difficult all the time/impossible	101 (10.8)	118 (12.6)	36 (5.3)	38 (5.6)	22 (15.4)	34 (23.4)	43 (38.7)	46 (41.1)

**Number of times visited a psychologist/counsellor (past 12 months)**								
0	549 (58.8)	529 (56.6)	436 (64.2)	428 (63.1)	64 (44.8)	55 (37.9)	49 (44.1)	46 (41.1)
1–6	292 (31.3)	312 (33.4)	187 (27.5)	198 (29.2)	57 (39.9)	65 (44.8)	48 (43.2)	49 (43.8)
≥7	92 (9.9)	94 (10.1)	56 (8.2)	52 (7.7)	22 (15.4)	25 (17.2)	14 (12.6)	17 (15.2)

**Current use of antidepressants**	190 (20.4)	226 (24.2)	92 (13.5)	120 (17.7)	54 (37.8)	58 (40.0)	44 (39.6)	48 (42.9)

**Frequency of internet use**								
Daily	904 (96.9)	910 (97.3)	662 (97.5)	661 (97.5)	139 (97.2)	142 (97.9)	103 (92.8)	107 (95.5)
Less than daily	29 (3.1)	25 (2.7)	17 (2.5)	17 (2.5)	4 (2.8)	3 (2.1)	8 (7.2)	5 (4.5)

a *Denominators may vary due to missing data. Anxiety symptom severity missing* n *= 287 (*n *= 134 intervention arm;* n *= 153 in control arm); mental health self-efficacy missing* n *= 280 (*n *= 137 in intervention arm;* n *= 143 in control arm); quality of life missing* n *= 184 (*n *= 92 in intervention arm;* n *= 92 in control arm); receiving benefit or disability support missing* n *= 138 (*n *= 68 in intervention arm;* n *= 70 in control arm). AQoL-8D = Assessment of Quality of Life Instrument. GAD-7 = 7-item Generalised Anxiety Disorder scale. MHSES = Mental Health Self-Efficacy Scale. PHQ-9 = 9-item Patient Health Questionnaire. SD = standard deviation.*

### 

#### Primary outcome

**Figure 2.**
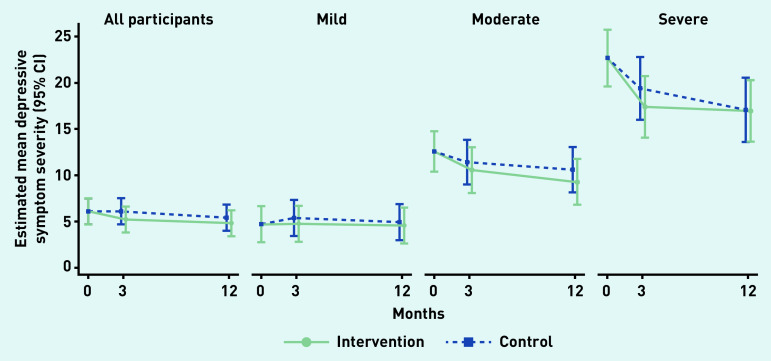
***Estimated mean depressive symptom severity (9-item Patient Health Questionnaire) with 95% confidence intervals for each trial arm, in total and by prognostic group and time-point.***

The estimated difference in mean depressive scores at 3 months was −0.88, favouring the intervention arm (95% confidence interval [CI] = −1.45 to −0.31) (see Table 2 and Figure 2), equivalent to a SMD of −0.16 (95% CI = −0.26 to −0.05). Findings were robust to different missing data assumptions (see Supplementary Appendix S4).

**Table 2.** Depressive symptom severity (PHQ-9) score according to trial arm, in total and stratified by prognostic group

	**All participants**		**Prognostic group**					

			**Minimal/mild**		**Moderate**		**Severe**	
			
	***n***	**Mean (SD)**	***n***	**Mean (SD)**	***n***	**Mean (SD)**	***n***	**Mean (SD)**
**3 months**								
Intervention	594	8.26 (6.02)	439	6.59 (5.04)	80	11.64 (5.51)	75	14.40 (6.39)
Control	668	9.16 (6.51)	483	7.29 (5.60)	112	12.69 (5.62)	73	16.10 (6.49)

**12 months**								
Intervention	563	7.77 (5.85)	421	6.33 (5.01)	74	10.53 (5.68)	68	13.69 (6.11)
Control	602	8.44 (6.19)	441	6.82 (5.26)	103	12.10 (6.14)	58	14.28 (6.64)

**Total**	1868	—	1357	—	288	—	223	—

		***P*-value**		***P*-value**		***P*-value**		***P*-value**

**3 months**								
Difference in mean outcome between arms (95% CI)^a^	−0.88	0.003	−0.62	0.04	−0.84	0.29	−1.98	0.06
	(−1.45 to −0.31)		(−1.21 to −0.03)		(−2.40 to 0.72)		(−4.00 to 0.04)	
Sensitivity analysis^b^	−0.87 (−1.43 to −0.30)	0.003	−0.62 (−1.21 to −0.04)	0.04	−1.08 (−2.66 to 0.49)	0.18	−2.16 (−4.20 to −0.12)	0.04
Sensitivity analysis^c^	—	—	—	—	—	—	−1.98 (−4.00 to 0.04)	0.06
CACE analysis^d^	—	—	—	—	—	—	−5.23 (−10.9 to 0.44)	0.07
SMD^a,e^	−0.16 (−0.26 to −0.05)	—	−0.18 (−0.36 to −0.01)	—	−0.36 (−1.02 to 0.31)	—	−0.56 (−1.12 to 0.01)	—

**12 months**								
Difference in mean outcome between arms (95% CI)^a^	−0.59 (−1.18 to 0.01)	0.05	−0.35 (−0.94 to 0.25)	0.26	−1.35 (−3.02 to 0.32)	0.11	−0.10 (−2.29 to 2.08)	0.93
Sensitivity analysis^b^	−0.57 (−1.16 to 0.02)	0.06	−0.34 (−0.93 to 0.25)	0.26	−1.47 (−3.14 to 0.21)	0.09	−0.19 (−2.43 to 2.04)	0.87
Sensitivity analysis^c^	—	—	—	—	—	—	−0.10 (−2.29 to 2.08)	0.93
CACE analysis^d^	—	—	—	—	—	—	−1.65 (−7.61 to 4.31)	0.59
SMD^a,e^	−0.10 (−0.21 to 0.002)	—	−0.10 (−0.28 to 0.08)	—	−0.58 (−1.29 to 1.14)	—	−0.03 (−0.64 to 0.58)	—

a Estimated difference in mean outcome between intervention and control arms using linear mixed-effects regression with random intercepts for individuals and adjusted for baseline outcome measure, general practice, time and prognostic group (for all partcipants only); mean outcome was constrained to be equal at baseline.

b Same as ^‘a’^, adjusted for factors associated with non-response at 3 and 12 months (age, sex, highest level of education, current employment status, hold a health care card, long term illness, live alone, self-rated health, manage on available income, number of times visited a psychiatrist or counsellor in past 12 months, and current use of antidepressants).

c Same as ^‘a’^, adjusted for imposed clustering by nurse in the intervention arm in the severe prognostic group only; 6 nurses (cluster size [range] 1 to 31, median = 14 patients). Estimated intra-cluster correlation for imposed clustering was zero.

d Adherence-adjusted analysis for severe group only (adherence = completed all eight sessions).

e SMD was calculated as the difference in means between arms and divided by the pooled SD at baseline for all participants (SD = 5.71); minimal/mild (SD = 3.39); moderate (SD = 2.34); and severe (SD = 3.56) group. CACE = complier average causal effect. PHQ-9 = 9-item Patient Health Questionnaire. SD = standard deviation. SMD = standardised mean difference.

#### Secondary outcomes

At 12 months, weak evidence supported a smaller intervention effect on depressive symptoms at 12 months overall (see Table 2 and Figure 2), but no evidence for a difference in mean anxiety symptom severity between trial arms (see Table 3 and Supplementary Appendix S4). At 3 months mean mental health self-efficacy was 1.39 points higher in the intervention arm compared to the control arm (95% CI = 0.31 to 2.46). There was no evidence of an overall difference in mean quality of life between trial arms, although within the moderate group, mean scores were 0.05 points higher in the intervention arm (95% CI = 0.01 to 0.09) compared to control arm.

**Table 3.** Estimated difference in mean between trial arms for secondary outcomes, in total and stratified by prognostic group

	**All participants**		**Prognostic group**					

			**Minimal / mild**		**Moderate**		**Severe**	
			
	**Difference in mean outcome (95% CI)^a^**	***P*-value**	**Difference in mean outcome (95% CI)^a^**	***P*-value**	**Difference in mean outcome (95% CI)^a^**	***P*-value**	**Difference in mean outcome (95% CI)^a^**	***P*-value**
**Anxiety symptom severity (GAD-7)**								
Total analysed, *n*	1780	—	1285	—	278	—	217	—
3 months	−0.43 (−0.99 to 0.12)	0.13	0.10 (−0.50 to 0.70)	0.74	−1.17 (−2.63 to 0.28)	0.11	−1.18 (−2.99 to 0.63)	0.20
12 months	−0.17 (−0.78 to 0.45)	0.59	0.0005 (−0.66 to 0.66)	0.99	0.13 (−1.55 to 1.80)	0.88	−0.13 (−2.19 to 1.94)	0.91

**Mental health self-efficacy (MHSES)**								
Total analysed, *n*	1779	—	1284	—	278	—	217	—
3 months	1.39 (0.31 to 2.46)	0.01	1.06 (−0.15 to 2.27)	0.09	2.35 (−0.22 to 4.91)	0.07	1.55 (−2.10 to 5.20)	0.41
12 months	0.87 (−0.43 to 2.17)	0.19	0.49 (−0.88 to 1.86)	0.48	1.10 (−2.53 to 4.73)	0.55	1.20 (−3.62 to 6.02)	0.63

**Quality of life (AQoL-8D)**								
Total analysed, *n*	1761	—	1270	—	277	—	214	—
3 months	0.011 (−0.005 to 0.027)	0.16	0.0005 (−0.017 to 0.019)	0.96	0.047 (0.007 to 0.088)	0.02	0.033 (−0.010 to 0.075)	0.13
12 months	0.013 (−0.007 to 0.033)	0.19	0.010 (−0.013 to 0.033)	0.39	0.014 (−0.039 to 0.067)	0.60	0.028 (−0.037 to 0.093)	0.40

a Estimated using linear mixed-effects regression with random intercepts for individuals and adjusted for baseline outcome measure, general practice, time, and prognostic group (for all participants only); mean outcome is constrained to be equal at baseline. AQoL-8D = Assessment of Quality of Life Instrument. GAD-7 = 7-item Generalised Anxiety Disorder scale. MHSES = Mental Health Self-Efficacy Scale.

### Adherence-adjusted analyses

Five (0.7%) of 679 intervention participants in the minimal/mild group completed at least one myCompass module, and eight (5.6%) of 143 participants in the moderate group completed the Worry and Sadness course in full (see Supplementary Appendix S4). Given the few completers in these groups, no further planned analyses were conducted.

In the severe group, 64 participants (57.7%) attended at least one collaborative care appointment and 30 (27.0%) of 111 participants attended all eight (see Supplementary Appendix S4). Participants who completed all eight collaborative care appointments had a 5.2-point greater reduction in mean PHQ-9 score at 3 months (95% CI = −10.9 to 0.44) compared to their control arm counterparts, equivalent to an SMD of −1.4 (95% CI = −3.0 to 0.12) (data not shown).

## DISCUSSION

### Summary

To the authors’ knowledge, this is the first RCT of a person-centred e-health platform supporting prognosis-based allocation of depression management in primary care. For the primary outcome, results favoured the intervention overall, although the effect size was small. Pre-specified adherence-adjusted analysis identified greater improvements associated with completion of collaborative care in the severe group. The intervention effect on depressive symptoms had diminished by 12 months, and few differences were observed on secondary outcomes.

### Strengths and limitations

Strengths include: individual randomisation and a primary outcome measure that allows comparison with international studies; successful recruitment to target with a balance of baseline characteristics between trial arms; primary outcome completion rates comparable to previous stepped depression care trials in primary care;^36^^–^^41^ and a pragmatic design that tested a model of care designed for scalability. However, low uptake of online management options limited the ability to complete planned analyses, and the wide availability of depression care in Australian primary care^42^ (see Supplementary Appendix S5) may have reduced the potential for the intervention to improve on usual care. The focus on depression may reduce generalisability to other mental health conditions and the authors did not assess symptom duration at enrolment, although a low threshold for eligibility was set and interventions were not disorder specific. Greater attrition than anticipated was observed and finally, the approach was limited to initial allocation only and intervention intensity was not adjusted according to participant response.

### Comparison with existing literature

Development of revolutionary new treatments for depression is considered unlikely.^43^ Instead, efforts to reduce the burden of disease have focused on better tailoring of existing interventions, leading to the development and testing of a range of stepped care approaches. While overall effect size at 3 months (−0.16) was lower than that reported in a stepped care meta-analysis (−0.34),^44^ CIs included the clinically relevant value of −0.24 proposed by Cuijpers and colleagues.^45^ Importantly, effect in the present study was achieved through delivery of a minimally time- and resource-intensive intervention in a routine setting across a large number of practices. Further, the meta-analysis by Cuijpers and colleagues^45^ assessed the effectiveness of stepped care in people meeting diagnostic criteria for depression whereas effect in the present study was observed in a heterogenous sample reflecting real-life primary care.^46^

Within prognostic groups, observed effect was smaller than that reported in previous RCTs of myCompass^32^ and This Way Up^47^^,^^48^ but similar to iCBT effect sizes in primary care settings.^49^^,^^50^ This is further compounded by the low rates of programme completion, although even if all participants completed their recommended programme, they had limited room for symptom improvement. Within the severe group, estimated effect size was consistent with previous trials of collaborative care,^25^^,^^51^^–^^54^ contributing to the growing literature showing that nurse-delivered collaborative care is both effective and acceptable in the management of depression.^55^ Participants who completed the full course of collaborative care reported substantial improvements, the effect size comparing favourably to that associated with antidepressants.^56^ Analysis of the characteristics of completers and their tailored management plans is underway to refine the intervention to enhance uptake and completion (manuscript in preparation).

This novel, theory-driven approach provides not only assessment but a prompt to reflect on priorities and motivation, aiming to empower patients to take ownership of their mental health care. Patients are triaged to care according to their predicted severity of depressive symptoms in 3 months’ time, rather than severity when first assessed. This approach incorporates broader determinants of poor mental health (for example, financial and physical health difficulties), which are critical to delivering comprehensive primary care, and recognises that mild and transitory depressive symptoms are prevalent and will often resolve without formal intervention. Current policy aims to redirect the minimal/mild group away from face-to-face services and towards lower intensity (including online) interventions, in line with clinical guidelines.^4^^,^^5^ However, present study findings suggest simply recommending these interventions, even when designed to activate patients towards uptake,^18^ was insufficient to encourage their use. This experience is not unique; research and policy interest in online interventions has not yet translated into their widespread use (and the multifaceted reasons for this are discussed elsewhere).^57^^–^^61^ This is a rapidly evolving field and emerging health, social, and economic levers may improve acceptability of online interventions, and thus the potential for an approach like Target-D to serve as an effective conduit. For instance, public health crises such as the COVID-19 pandemic may necessitate greater engagement with online programmes^62^ due to overburdened health systems and a pressing need to efficiently triage people to mental health care without lengthy consultation in general practice.

### Implications for research and practice

Mental health remains the predominant issue managed in primary care,^63^ despite substantial and sustained investment. Worldwide, health systems face the challenge of ensuring that investments are well targeted to optimise patient outcomes and experiences of care. It is likely that improvements in mental health care will be incremental and gained by ongoing optimisation of promising approaches. The Target-D person-centred, e-health platform, which can quickly and easily triage and tailor depression care to severity prognosis, is a promising component of stepped mental health care. The authors present the cost-effectiveness of this approach elsewhere.^64^ While the trial was not set up to test the effectiveness of the Target-D platform and matched management options in preventing disorder onset, this may be an avenue for future research. Alternatively, an option for implementation may be to offer the Target-D platform to all patients but provide matched management options only to the moderate and severe groups where the potential for improvement is greater. Findings also support further research into how to optimise uptake, particularly of low intensity services.
